# Commentary: Type I Interferon Response Is Mediated by NLRX1-cGAS-STING Signaling in Brain Injury

**DOI:** 10.3389/fnmol.2022.947542

**Published:** 2022-06-27

**Authors:** Cali M. McEntee, Thomas J. LaRocca

**Affiliations:** ^1^Department of Health and Exercise Science, Colorado State University, Fort Collins, CO, United States; ^2^Center for Healthy Aging, Colorado State University, Fort Collins, CO, United States

**Keywords:** cGAS-STING, traumatic brain injury, interferon, neuroinflammation, cytosolic dsDNA

Neuroinflammation is a central feature of traumatic brain injury (TBI; Smith et al., 2013; Lozano et al., 2015), and it is also implicated in neurodegeneration (Chen et al., 2016; Hong et al., 2016). In fact, studies have linked a history of TBI and future development of neurodegenerative diseases (Surgucheva et al., 2014; Gardner and Yaffe, 2015; Wilson et al., 2017), making the understanding of TBI-related neuroinflammation a high priority. A key mechanism underlying neuroinflammation with TBI is the activation of the cyclic GMP-AMP and Stimulator of Interferon Genes (cGAS-STING) pathway.

cGAS-STING is activated in response to cytosolic double-stranded DNA (dsDNA; Shu et al., 2014; Hopfner and Hornung, 2020), which may originate from mitochondrial or nuclear DNA (Glück et al., 2017; Matsui et al., 2021) due to cell damage/stress (Dunphy et al., 2018; Akbari et al., 2021). Activated cGAS-STING (*via* STING phosphorylation) upregulates transcription factors that stimulate interferons (IFNs), interferon-stimulating genes (ISGs), and pro-inflammatory cytokines (Decout et al., 2021). STING phosphorylation can be inhibited by nucleotide-binding oligomerization domain leucine-rich repeat containing X1 (NLRX1; Guo et al., 2016), but until recently, the role of cGAS-STING activation and NLRX1 in TBI *in vivo* was unknown.

Fritsch et al. (2022) addressed this gap in knowledge using an *in vivo* model of TBI. Mice were subjected to a controlled cortical impact (CCI) injury, and the authors found that CCI injury increased IFN and pro-inflammatory transcripts, including STING transcripts, which remained elevated 24 h after injury. They also showed that cGAS-STING activation from CCI injury coincided with increased presence of cytosolic mitochondrial dsDNA (but not nuclear dsDNA). When the authors repeated their experiments in homozygous cGAS and STING knockout mice, they found reduced IFN and pro-inflammatory transcript accumulation, as well as less brain tissue damage and neuronal apoptosis, indicating that cGAS and STING are required for the detrimental effects of TBI. Lastly, they showed that CCI injury in NLRX1 knockout mice increased STING phosphorylation and reduced IFN and pro-inflammatory transcripts. Overall, their data suggest that cGAS-STING may be an important contributor to neuroinflammation with TBI.

A strength of this study is the *in vivo* model of TBI *via* CCI injury, a common pre-clinical model in which impact depth, velocity, dwell time, and impact tip size are standardized (Osier and Dixon, 2016). This ensures that all mice receive the same TBI, and it allows the contralateral side of the brain to be used as an “in-mouse” control. In fact, in Supplementary Figure 3, Fritsch et al. showed no difference between sham (no injury) ipsilateral and CCI injury contralateral cortices (Fritsch et al., 2022). Thus, any observed changes were the result of TBI-related effects and not “inter-mouse” differences. Also, in their CCI ipsilateral vs. contralateral comparisons, the authors included a range of gene expression measurements (e.g., of ISGs) that have been previously documented in measuring cGAS-STING activation (Willemsen et al., 2021), as well as new histological outcomes (e.g., lesion volume) associated with cGAS-STING activation.

Despite the strengths of the work by Fritsch et al., this study also raises some important questions. Perhaps the most important question is whether nuclear or mitochondrial cytosolic dsDNA is the key contributor to cGAS-STING activation with TBI. For example, studies in humans have shown that nuclear dsDNA is implicated in TBI (Schwab et al., 2019) and cGAS-STING signaling (Li and Chen, 2018). Fritsch et al. did measure cytosolic HMGB1 (Figure 2F, Fritsch et al., 2022), a nuclear dsDNA protein reported to be involved in TBI (Paudel et al., 2018), but they saw no difference between ipsilateral and contralateral cortices after CCI injury. However, this is only one marker of nuclear dsDNA, and its absence does not definitively confirm that nuclear dsDNA is not involved in cGAS-STING activation. To address this issue, future studies could utilize more generic markers of nuclear dsDNA, like anti-dsDNA antibodies (Zhou et al., 2021) combined with markers of DNA damage (Glück et al., 2017), and/or nuclear DNA-specific probes could be used to prove that nuclear dsDNA is not present in the cytosol. Perhaps even better, immunoprecipitation of nucleic acids bound to cGAS could be used to test the TBI/mitochondrial dsDNA hypothesis. Pre-treatment with compounds that protect mitochondria against stress/injury, like MitoQ, could also be used to further confirm the role of mitochondrial dsDNA in cGAS-STING activation in TBI, as mitochondrial dsDNA in the cytosol is often the result of damage to the mitochondria (Chung et al., 2019). Similar studies with compounds that modulate nuclear permeability could address the relative role of nuclear dsDNA. Experiments like these would more convincingly demonstrate that mitochondrial vs. nuclear dsDNA accumulation activates cGAS-STING in TBI. Admittedly, the distinction between nuclear and mitochondrial dsDNA would not matter once cGAS-STING signaling is activated, but identifying the source of these cytosolic dsDNAs could be important for “upstream” therapeutic approaches.

Another important point is that impaired behavioral function is commonly associated with TBI in mice and humans (Gorgoraptis et al., 2019; Xu et al., 2021), but behavioral testing was not performed in this study. Such data would connect the molecular and pathological findings with CCI injury to physiological dysfunction often seen with TBI, and demonstrate that inhibiting cGAS-STING signaling may be a viable therapeutic strategy. Fritsch et al. did mention that behavioral changes in response to CCI-induced TBI were previously shown (Barrett et al., 2020) and therefore, they investigated motor dysfunction instead. However, the cited study used an IFNβ homozygous knockout mouse and not cGAS and STING knockouts (Barrett et al., 2020). IFN activation, specifically of IFNβ, can result from signaling *via* pathways other than cGAS-STING, like the RIG-I/MDA5 pathway (which responds to dsRNA; Dhir et al., 2018), and therefore, it cannot be concluded that behavioral impairments seen with an IFNβ knockout would be similar to those in cGAS and/or STING knockouts. Furthermore, Fritsch et al. did not demonstrate a direct link between cGAS-STING, cytokines/IFNs, and brain pathology, and cGAS-STING could modulate other pathways that may influence pathology, like autophagy (Liu et al., 2018), apoptosis (Cerboni et al., 2017), and tau phosphorylation (*via* cGAS targets like TBK1; Abreha et al., 2021).

Finally, Fritsch et al. found greater gene expression of cGAS and STING in microglia compared to other brain cells, suggesting that microglia may be central to cGAS-STING activation (Supplementary Figure 5, Fritsch et al., 2022), which is an important observation consistent with the central role of microglia in neuroinflammation (Shao et al., 2022). However, others have also shown that astrocytes are involved in TBI-related responses (Burda et al., 2016; Michinaga and Koyama, 2021) and cGAS-STING activation (Jeffries and Marriott, 2017), and the current data do not rule out the contribution of other glial cells—especially since cell isolation protocols themselves can contribute to inflammatory microglial activation (Cadiz et al., 2022) and the authors did not report on cGAS-STING levels in adherent cells other than microglia. In addition to studying other cells like astrocytes, future studies could leverage single-cell sequencing approaches (e.g., single-cell RNA-seq) to confirm the importance of microglia and/or other cell types in cGAS-STING activation with TBI.

To conclude, Fritsch et al. have nicely documented the importance of cGAS-STING activation in TBI-related neuroinflammation using cGAS and STING knockouts. However, future studies could be conducted to confirm their findings and provide important insight on specific mechanisms and potential therapeutic strategies related to TBI-induced neuroinflammation.

## Author Contributions

CM wrote the manuscript. CM and TL revised the manuscript. Both authors approved the manuscript for submission.

## Funding

TL and CM were supported by grants from the National Institute on Aging (AG060302 and AG070562).

## Conflict of Interest

The authors declare that the research was conducted in the absence of any commercial or financial relationships that could be construed as a potential conflict of interest.

## Publisher's Note

All claims expressed in this article are solely those of the authors and do not necessarily represent those of their affiliated organizations, or those of the publisher, the editors and the reviewers. Any product that may be evaluated in this article, or claim that may be made by its manufacturer, is not guaranteed or endorsed by the publisher.
